# Comparison of bilateral implantation of an extended depth of focus lenses and a blend approach of extended depth of focus lenses and bifocal lenses in cataract patients

**DOI:** 10.1186/s12886-023-03228-1

**Published:** 2023-11-21

**Authors:** Tianxu Xiong, Hao Chen, Wei Fan

**Affiliations:** Department of Ophthalmology, West China Hospital of Sichuan University, 610041 Chengdu, Sichuan Province China

**Keywords:** Cataract, Presbyopia corrected intraocular lens, Micro monovision, Blend

## Abstract

**Background:**

To compare the visual outcomes, spectacle independent rate and stereopsis in patients who underwent bilateral implantation of extended depth of focus (EDOF) intraocular lens (IOL), or a blend approach of EDOF and bifocal IOL.

**Methods:**

A total of 60 cataract patients, who were scheduled for phacoemulsification and intraocular lens implantation in both eyes in West China Hospital of Sichuan University, were enrolled and divided into Micro monovision group(-0.5D~-1.0D), Non-micro monovision group (< 0.5D) with Symfony IOL, and Mixed group with Symfony and ZMB00 IOLs. Three months postoperatively, we compared the visual acuity, modulation transfer function (MTF), defocus curve, stereopsis, spectacle independence, and photic phenomena among the three groups.

**Results:**

Compared to the Non-micro monovision group (UNVA: 0.07 ± 0.04), Micro monovision group (UNVA: 0.00 ± 0.07, *P* < 0.001) and Mixed group (UNVA: -0.02 ± 0.06, *P* < 0.001) showed improvement in binocular uncorrected near visual acuity (UNVA). Additionally, Mixed group exhibited lower MTF10 (MTF10: 0.38 ± 0.24) and point spread function (PSF: 0.192 ± 0.269) results in their non-dominant eye compared to both Micro monovision group (MTF10: 0.56 ± 0.21, *P* = 0.027; PSF: 0.417 ± 0.282, *P* = 0.034) and Non-micro monovision group (MTF10: 0.55 ± 0.19, *P* = 0.038; PSF: 0.408 ± 0.285, *P* = 0.003). Spectacle independence for near vision were higher in both the Micro monovision (45%) and Mixed (55%) group compared to the Non-micro monovision group (40%). The Mixed group also reported higher incidence of photic phenomena (25%). However, there were no significant differences in stereoscopic function among the three groups.

**Conclusion:**

Both micro monovision and mix-and-match methods can help patients to obtain better visual outcomes at different distances. Mix-and-match method has better near visual acuity, while micro monovision method has better intermediate visual acuity. Non-micro monovision methods will affect patients’ near vision outcomes. Binocularly implanted EDOF IOL has better contrast sensitivity.

**Clinical trial registration:**

Registration date:11/07/2023. Trial registration number: ChiCTR2300073433. Trial registry: West China Hospital of Sichuan University retrospectively registered.

## Background

Cataract surgery has evolved into lens-based refractive surgery with rapid intraocular lens (IOL) optical technology advances and improvements in equipment and techniques [1]. Helping patients obtain the better postoperative visual outcomes has become the primary goals of modern cataract surgery [2]. To achieve spectacle independence, several intraocular lenses (IOLs) are used to correct presbyopia, including multifocal, accommodative and the Extended Depth of Focus (EDOF) IOLs. Among these, EDOF IOL is a new type of presbyopia correction IOL.

The TECNIS Symfony EDOF IOL (Johnson & Johnson Vision, Santa Ana, USA) incorporates a specialized diffractive echelette design. This unique design effectively disperses light, resulting in an elongated focus and ensuring a seamless and unobstructed range of vision. In comparison to bifocal and trifocal IOLs, EDOF IOLs have limited visual results at near distance, but have a lower incidence of photic phenomena [3]. However, clinical studies with TECNIS Symfony IOLs also showed the visual results are less satisfactory for near distances [4–5]. Therefore, there are some methods to solve this problem. Among them, micro monovision and mix and match methods are usually used. Although these methods can help patients obtain good visual outcomes, there is a few studies comparing the two methods [6–9]. Therefore, the purpose of our study was to compare the visual outcomes at different distances, depth of focus, stereopsis, optical quality in three groups, which include bilateral implantation of an EDOF IOL (Micro monovision group and Non-micro monovision group) and a blend of EDOF and bifocal IOL (Mixed group).

## Methods

### Patients

This study is a prospective cohort focusing on patients with bilateral age-related cataract who were scheduled for phacoemulsification and intraocular lens implantation from May 2022 to June 2023 in Dept. of ophthalmology in West China Hospital of Sichuan University. All patients were offered participation during this period and 9 patients withdrew before the end of the study due to geographical inconvenience. The study was approved by the medical ethics committee of West China Hospital and conducted in accordance with the principles of the Declaration of Helsinki. Patients were divided into three groups based on their own vision requirements at different distances and their choice of IOLs. According to the preoperative target refraction and the actual refractive result at 3 months after operation, patients with both eyes implanted with Symfony IOL were divided into Micro monovision group (difference of spherical equivalent refraction of both eyes was between − 0.5D and − 1.0D) and Non-micro monovision group (difference of spherical equivalent refraction of both eyes was <0.5D. Patients implanted with Symfony IOL in the dominant eye and a bifocal IOL (ZMB00, Johnson & Johnson Vision, Santa Ana, USA) in the nondominant eye were included in the Mixed group.

We recruited patients with bilateral age-related cataract who wished to be free of glasses. Patients were included if they were over 18 years of age, had a postoperative residual corneal astigmatism ≤ 1.0D, a natural pupil diameter between 3.0 and 5.5 mm in the dark room, and an angle κ less than 0.5 mm or half the diameter of the central refractive optical zone of the IOL. Patients with any of the following conditions were excluded from the study: microphthalmia, pathological myopia, obvious pupil abnormalities, severe corneal lesions, chronic uveitis, glaucoma, progressive retinal diseases, severe optic nerve diseases, history of ocular trauma or ocular surgery which may influence the calculation of IOL power or postoperative vision, professional drivers and frequent nighttime drivers, severe ocular surface disease, and patients incapable of cooperating with exams or follow-up. All patients must sign a written informed consent form and are informed of the surgical and follow-up plan, possible complications and precautions.

### Procedures

The intraocular lens power was calculated according to the data of IOL master 700. The data were measured repeatedly for at least two times to ensure their accuracy. The calculation formulas were selected according to the length of the patient’s ocular axis, and the refractive power of the intraocular lens was determined after comparison with multiple formulas including Barrett universal II, Haigis, SRK-T, Holladay 2, and Kane. The Symfony IOL power was aimed at emmetropia in the dominant eye and minimal residual myopia (-0.5 ~ 0.75D) or emmetropia in the non-dominant eye in both the micro-monovision and non-micro-monovision groups. The target refraction for both eyes in the mixed group was considered to be emmetropia.

Preoperative examinations included IOL Master 700(Carl Zeiss Meditec AG, Jena, Germany), corneal topography (Tomey, Japan), iTrace (Tracey Technologies Corp., Houston, TX, USA) and optical coherence tomography (HEIDELBERG, Germany) and measurement of the dominant eyes. Three months after the second eye surgery, the main observational outcomes include uncorrected distance visual acuity (UDVA) at 5 m, uncorrected intermediate visual acuity (UIVA) at 80 cm, uncorrected near visual acuity (UNVA) at 40 cm, monocular and binocular defocus curve from + 2.0D to -4.0D (0.5 D steps), MTF and PSF (scan diameter 3.0 mm), VF-14 questionnaire and stereopsis examination.

### Intraocular lenses and surgery

The TECNIS Symfony IOL (Johnson & Johnson Vision, Santa Ana, USA) is a single-piece, aspheric EDOF IOL. The optical zone is 6.0 mm. It has a diffractive posterior surface which has echelette design to form an elongated focus.

The TECNIS ZMB00 (Johnson & Johnson Vision, Santa Ana, USA) is a single-piece, aspheric bifocal lens. The optical zone is 6.0 mm. It has a diffractive multifocal posterior surface which designed to provide both near and distance vision, with a near power of + 4.0 D.

The same experienced surgeon performed phacoemulsification and implantation of TECNIS Symfony IOL or ZMB00 IOL using the Stellaris phacoemulsification system (Bausch&Lomb, Rochester NY, USA) in all patients. The main incision size is 2.0 mm. Surgical navigation (Calisto Eye, Carl Zeiss Meditec AG, Jena, Germany) was used in 19 eyes to correct a preoperative corneal astigmatism of > 0.75D with a steep axis corneal incision (2.75 mm). The interval between binocular surgeries is 1 week.

### Statistical analysis

The statistical software SPSS 26.0 (IBM Corp., Armonk, NY) was utilized for data analysis. The mean ± SD was used to report all values. The normality of data samples was evaluated with the Kolmogorov–Smirnov test. To compare continuous variables among the three groups, ANOVA or non-parametric Kruskal–Wallis tests were used, depending on the normal distribution of the data. The chi-square test was used to compare classification variables among the three groups. Turkey’s and Bonferroni’s multiple comparisons test were used to adjust the *p* value for between group comparisons. Additionally, sub-group comparisons were conducted using independent t-tests and non-parametric tests. *P* value less than 0.05 was considered statistically significant.

## Results

Sixty patients were recruited for the study, with 20 patients in each group. The axial length of Mixed group was longer than that of Micro monovision group and Non-micro monovision group (*P* = 0.033, *P* < 0.001), and the ocular axis of Micro monovision group was longer than that of Non-micro monovision group (*P* = 0.042). The depth of anterior chamber in Mixed group was deeper than that in Non-micro monovision group (*P* = 0.016). The preoperative corneal astigmatism in Mixed group was higher than that in Micro monovision group (*P* < 0.001). Table 1 shows further details of preoperative data for the three groups.

**Table 1:** Baseline of three groups

	Micro monovision group	Non-micro monovision group	Mixed group	*P*	Adjusted *P* (Micro monovision vs. Non-micro monovision)	Adjusted *P* (Micro monovision vs. Mixed)	Adjusted *P* (Mixed vs. Non-micro monovision)
Male: Female	8:12	8:12	4:16	0.301 ^a^			
Age	58.20 ± 8.87	62.75 ± 8.92	57.85 ± 9.26	0.069 ^c^			
Axial length	24.73 ± 2.16	23.72 ± 1.60	25.64 ± 1.57	0.000* ^c^	0.042*	0.033*	0.000*
Anterior chamber depth	3.24 ± 0.49	3.13 ± 0.57	3.38 ± 0.32	0.019* ^c^	0.836	0.262	0.016*
Corneal curvature	43.60 ± 1.47	43.88 ± 1.41	44.12 ± 1.28	0.368 ^c^			
corneal astigmatism	0.48 ± 0.32	0.65 ± 0.36	0.78 ± 0.32	0.001* ^b^	0.076	0.000*	0.182
Corneal HOA of dominant eye(3 mm pupil)	0.081 ± 0.049	0.079 ± 0.040	0.105 ± 0.058	0.285 ^c^			
Corneal HOA of non-dominant eye(3 mm pupil)	0.077 ± 0.040	0.076 ± 0.040	0.088 ± 0.051	0.745 ^c^			
Corneal SphAb of dominant eye(3 mm pupil)	0.215 ± 0.056	0.236 ± 0.087	0.212 ± 0.078	0.535 ^b^			
Corneal SphAb of non-dominant eye(3 mm pupil)	0.212 ± 0.055	0.213 ± 0.082	0.210 ± 0.088	0.992 ^b^			
Angle α of dominant eye	0.388 ± 0.149	0.424 ± 0.163	0.272 ± 0.157	0.008* ^b^	0.748	0.056	0.009*
Angle α of non-dominant eye	0.347 ± 0.140	0.401 ± 0.157	0.261 ± 0.163	0.020* ^c^	1.000	0.144	0.020*
Angle κ of dominant eye	0.255 ± 0.144	0.328 ± 0.144	0.201 ± 0.145	0.017* ^c^	0.431	0.490	0.013*
Angle κ of non-dominant eye	0.269 ± 0.116	0.299 ± 0.174	0.219 ± 0.116	0.192 ^b^			

a: chi-square test. Bonferroni’s multiple comparisons test was used to adjust the *p* value for between group comparisons. b: one-way ANOVA test. Turkey’s multiple comparisons test was used to adjust the *p* value for between group comparisons. c: nonparametric test. Bonferroni’s multiple comparisons test was used to adjust the *p* value for between group comparisons. *: *P* < 0.05

### Visual outcomes

Table 2 showed the uncorrected visual acuity at 3 months. No statistically significant difference in binocular UDVA was observed among the three groups. (*P* = 0.143). Although the binocular UIVA in Micro monovision group was better than that in Non-micro monovision group and Mixed group (*P* = 0.010, *P* = 0.003), the mean UIVA of the three groups were all better than logMAR 0. Binocular UNVA showed a significant improvement in both the monovision and mixed groups compared to the non-micro monovision group. (*P* < 0.001).

**Table 2:** Uncorrected distance, intermediate, and near visual acuity at 3 months postoperatively (logMAR)

		Micro monovision group	Non-micro monovision group	Mixed group	*P*	Adjusted *P* (Micro monovision vs. N Non-micro monovision)	Adjusted *P* (Micro monovision vs. Mixed)	Adjusted *P* (Mixed vs. Non-micro monovision)
UDVA	dominant eye	0.01 ± 0.09	0.02 ± 0.06	0.00 ± 0.05	0.469^a^			
	non-dominant eye	0.12 ± 0.13	0.04 ± 0.06	0.02 ± 0.09	0.134^a^			
	binoculus	-0.02 ± 0.07	-0.01 ± 0.05	-0.06 ± 0.07	0.143^a^			
UIVA	dominant eye	-0.03 ± 0.04	0.01 ± 0.06	-0.01 ± 0.06	0.769^a^			
	non-dominant eye	-0.03 ± 0.05	0.00 ± 0.04	0.19 ± 0.08	0.000^a^*	0.332	0.000*	0.000*
	binoculus	-0.06 ± 0.04	-0.05 ± 0.04	-0.03 ± 0.06	0.001^a^*	0.010*	0.003*	1
UNVA	dominant eye	0.15 ± 0.09	0.16 ± 0.07	0.17 ± 0.12	0.118^a^			
	non-dominant eye	0.02 ± 0.08	0.11 ± 0.06	0.00 ± 0.08	0.000^a^*	0.000*	1.000	0.000*
	binoculus	0.00 ± 0.07	0.07 ± 0.04	-0.02 ± 0.06	0.000^a^*	0.000*	1.000	0.000*

a: nonparametric test. Bonferroni’s multiple comparisons test was used to adjust the *p* value for between group comparisons. *: *P* < 0.05

### Defocus curves

**Fig. 1:**
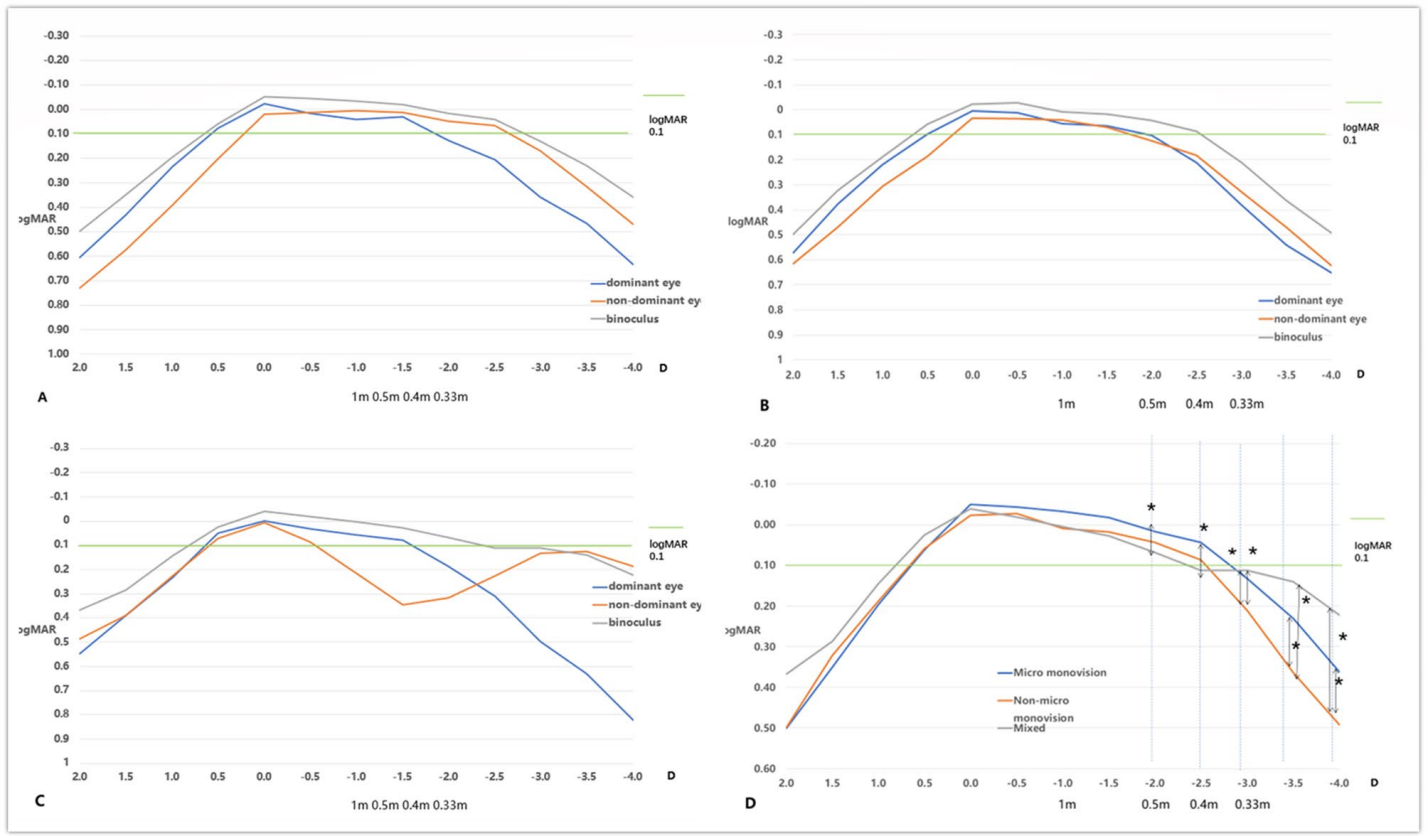
Three months post-op defocus curves (**A**: micro monovision group, **B**: non-micro monovision group, **C**: mixed group, **D**: Comparison of bilateral defocus curve)

The postoperative monocular and binocular defocus curves are shown in Fig. 1. The bilateral defocus curves of three groups all showed a smooth platform area below logMAR 0.1, the Micro monovision group is 0.50~ -2.75D, the Non-micro monovision group is 0.50~ -2.50D, and the Mixed group is 0.30~ -3.50D (Fig. 1A, B, C. When comparing the defocus curves among the three groups(Fig. 1D), there was no significant difference among three groups between 2~ -1.5D. However, between – 1.5 ~ − 2.5D, the Micro monovision group had best visual outcomes, the Mixed group had worst visual outcomes; between − 2~ -2.5D, the Micro monovision group was better than the Mixed group (*P*-2 = 0.042, *P*-2.5 = 0.021).; between – 2.5 ~ − 4.0D, the Mixed group had best visual outcomes, the Non-micro monovision group had worst visual outcomes; at – 3.0~ -3.5D, the Mixed group and Micro monovision group were better than the Non-micro monovision group (*P*-3 = 0.021, 0.047, *P*-3.5 < 0.001, 0.026); at – 4.0D, the Mixed group was better than Non-micro monovision group (*P* < 0.001).

### Stereoscopic function

The postoperative stereoscopic function is shown in Table 3. There was no significant difference in stereoscopic function among the three groups.

**Table 3:** Comparison of postoperative stereopsis in three groups

	Micro monovision group	Non-micro monovision group	Mixed group	*P*
stereopsis(”)	71.75 ± 36.24	69.44 ± 50.17	82.33 ± 54.75	0.588 ^a^

a: nonparametric test

### MTF and PSF

The postoperative MTF and PSF are shown in Table 4. The MTF and PSF of dominant eye in three groups had no significant difference. The MTF AVG height, MTF10, MTF20, MTF30 and PSF of non-dominant eye in mixed group were lower than those of Micro monovision group and Non-micro monovision group. There were significant differences in MTF10 and PSF between Mixed group and Micro monovision group (*P*_MTF_=0.027, *P*_PSF_=0.034) and between Mixed group and Non-micro monovision group (*P*_MTF_=0.038, *P*_PSF_=0.003). Besides, there was significant difference in MTF AVG height between Mixed group and Micro monovision group(*P* = 0.039).

**Table 4:** Comparison of postoperative MTF and PSF in three groups

		Micro monovision group	Non-micro monovision group	Mixed group	*P*	Adjusted *P* (Micro monovision vs. Non-micro monovision)	Adjusted *P* (Micro monovision vs. Mixed)	Adjusted *P* (Mixed vs. Non-micro monovision on)
MTF Avg Height	dominant eye	0.49 ± 0.18	0.48 ± 0.12	0.53 ± 0.17	0.544 ^b^			
	non-dominant eye	0.50 ± 0.17	0.48 ± 0.14	0.36 ± 0.19	0.027 * ^b^	0.968	0.039*	0.068
MTF10	dominant eye	0.56 ± 0.23	0.56 ± 0.17	0.62 ± 0.20	0.322 ^a^			
	non-dominant eye	0.56 ± 0.21	0.55 ± 0.19	0.38 ± 0.24	0.016 ^b^*	0.990	0.027*	0.038*
MTF20	dominant eye	0.34 ± 0.22	0.29 ± 0.15	0.37 ± 0.18	0.389 ^b^			
	non-dominant eye	0.33 ± 0.20	0.31 ± 0.16	0.21 ± 0.28	0.057 ^a^			
MTF30	dominant eye	0.25 ± 0.18	0.20 ± 0.10	0.26 ± 0.15	0.478 ^b^			
	non-dominant eye	0.24 ± 0.17	0.22 ± 0.19	0.15 ± 0.14	0.056 ^a^			
PSF	dominant eye	0.373 ± 0.264	0.369 ± 0.258	0.425 ± 0.277	0.494 ^a^			
	non-dominant eye	0.417 ± 0.282	0.408 ± 0.285	0.192 ± 0.269	0.009 ^a^*	1.000	0.034*	0.003*

a: nonparametric test. Bonferroni’s multiple comparisons test was used to adjust the *p* value for between group comparisons. b: one-way ANOVA test. Turkey’s multiple comparisons test was used to adjust the *p* value for between group comparisons. *: *P* < 0.05

### Spectacle independence rate

Table 5 shows the outcomes of the postoperative spectacle independence and the incidence of postoperative photic phenomena. For distance, intermediate and daily near vision, the majority of patients in all three groups achieved complete spectacle independence. However, when the patients of the three groups completed the meticulous near task, there were only 9 (45%) in the Micro monovision group, 8 (40%) in the Non-micro monovision group and 11 (55%) in the Mixed group did not need to wear glasses. There were 1 (5%) in Micro monovision group, 1 (5%) in Non-micro monovision group and 3 (25%) in mixed group complain about the dysphotopsia.

**Table 5:** Comparison of postoperative spectacle independence and dysphotopsia in three groups

				Micro monovision group	Non-micro monovision group	Mixed group	*P*	Adjusted *P* (Micro monovision and Non-micro monovision)		Adjusted *P* (Micro monovision and Mixed)		Adjusted *P* (Mixed and Non-micro monovision	
meticulous near task				9/20	8/20	11/20	0.000* ^a^		0.000*		1.000		0.000*
daily near task				20/20	20/20	20/20							
Intermediate task				20/20	20/20	20/20							
Distance task				19/20	19/20	19/20	1.000 ^b^						
dysphotopsia				1/20	1/20	5/20	0.000* ^b^				0.000*		0.000*

a: chi-square test. Bonferroni’s multiple comparisons test was used to adjust the *p* value for between group comparisons. *: *P* < 0.05

### Sub-group analysis

Further sub-group analysis in the Micro monovision group showed that there were 5 patients’ difference of spherical equivalent refraction of both eyes was between 0.75-1D, and 15 patients were between 0.50-0.75D. Although the statistical difference cannot be compared due to the small number of people in the subgroup analysis, the binocular UNVA in the 0.75 ~ 1D subgroup was better than that in the 0.5 ~ 0.75D subgroup. Meanwhile, the UDVA of non-dominant eyes and binocular stereopsis were not affected. More details are shown in Table 6.

**Table 6:** Sub-group comparison in micro monocular group

		0.75 ~ 1D (n = 5)	0.5 ~ 0.75D (n = 15)	*P*
Age		59 ± 8.26	57.21 ± 9.15	0.675 ^a^
Dominant eye	UNVA	0.15 ± 0.10	0.15 ± 0.19	0.977 ^a^
	UIVA	-0.06 ± 0.04	-0.03 ± 0.04	0.156 ^b^
	UDVA	0.00 ± 0.06	0.01 ± 0.07	0.964 ^b^
Non-dominant eye	UNVA	0.00 ± 0.06	0.03 ± 0.09	0.687 ^b^
	UIVA	-0.05 ± 0.04	-0.03 ± 0.05	0.559 ^b^
	UDVA	0.20 ± 0.16	0.09 ± 0.1	0.082 ^a^
Binoculus	UNVA	-0.02 ± 0.04	-0.003 ± 0.05	0.754 ^b^
	UIVA	-0.08 ± 0.00	-0.06 ± 0.04	0.319 ^b^
	UDVA	-0.01 ± 0.07	-0.02 ± 0.07	0.813 ^a^
Stereoscopic vision		53.8 ± 12.6	73.5 ± 40.33	0.391 ^b^
Pupil size under darkroom		4.43 ± 0.64	4.58 ± 0.96	0.612 ^a^
corneal astigmatism	dominant eye	0.46 ± 0.21	0.42 ± 0.23	0.314 ^a^
	Non-dominant eye	0.44 ± 0.30	0.47 ± 0.38	0.178 ^a^
Corneal SphAb	dominant eye	0.18 ± 0.03	0.18 ± 0.08	0.928 ^a^
	Non-dominant eye	0.19 ± 0.04	0.16 ± 0.08	0.316 ^a^

a: Independent sample t-test, b: nonparametric test

In the non-micro monovision group, 8 patients had binocular UNVA < LogMAR 0.1, and 12 patients had binocular UNVA ≥ LogMAR 0.1. Compared with the two subgroups, patients with better binocular UNVA were younger. Besides, difference of spherical equivalent refraction of both eyes was nearly 0.2D in binocular UNVA < LogMAR 0.1 sub-group. More details are shown in Table 7.

**Table 7:** Sub-group comparison in non-micro monovision group

	UNVA < LogMAR 0.1 (n = 8)	UNVA ≥ LogMAR 0.1 (n = 12)	*P*
Age	56.87 ± 7.86	63.87 ± 9.11	0.034 ^b^
Ocular axis	24.15 ± 2.18	23.70 ± 1.30	0.670 ^b^
Preoperative corneal astigmatism	0.49 ± 0.28	0.64 ± 0.33	0.168 ^a^
Postoperative corneal astigmatism	0.53 ± 0.26	0.58 ± 0.29	0.574 ^a^
Spherical equivalence of dominant eye	-0.28 ± 0.16	-0.20 ± 0.16	0.392 ^b^
Spherical equivalence of non-dominant eye	-0.44 ± 0.26	-0.24 ± 0.24	0.076 ^b^
Pupil size under darkroom	4.35 ± 0.81	4.27 ± 0.77	0.697 ^a^
Corneal SphAb of dominant eye	0.212 ± 0.08	0.195 ± 0.082	0.640 ^a^
Corneal SphAb of non-dominant eye	0.195 ± 0.086	0.182 ± 0.123	0.524 ^a^

a: Independent sample t-test, b: nonparametric test

## Discussion

Presbyopia correction IOL is one of the most important methods to correct presbyopia after cataract surgery. Presbyopia correction IOLs can be divided into multifocal IOL, EDOF IOL and accommodating IOL [2]. Because of the small adjustment range of accommodating IOL and the gradual loss of adjustment function in long-term follow-up, this IOL has not been widely used [10]. At present, the most commonly used presbyopia correction IOLs are multifocal IOL and EDOF IOL. Multifocal IOLs have 2 or 3 focal points, which are used for distance and near vision, or distance, intermediate, and near vision. The appearance of trifocal IOL improved intermediate vision compared with bifocal IOL [11]. EDOF IOL is essentially equivalent to a single focus IOL because it has only one extended focus. However, its extended focus is limited by the current technology and can only ensure distance and intermediate vision, and the near vision is limited [12].

Since presbyopia correction IOLs have different advantages and disadvantages, there are various choices in helping patients correct presbyopia after surgery. Because of the better contrast sensitivity in EDOF IOL, how to improve the near vision has become a problem to be discussed in many studies, including this one. Previous studies have proved that the micro monovision and mix and match can improve the near vision. The study of Ota et al. [13] showed that minimal residual myopia(-0.5D) in the nondominant eye and Non-micro monovision in the dominant eye can improve near vision compared with binocular Non-micro monovision. The study of Sri Ganesh et al. [14] showed that − 0.75D residual myopia can also obtain good near vision and have good tolerance to anisometropia. According to the research of song et al. [15], the EDOF IOL mixed with + 3.25D bifocal IOL group has better distance and intermediate vision than the bilateral implanted trifocal IOL group, but the near vision is not as good as trifocal IOL group. Acar et al. [16] showed that there was no statistical difference in the distance, intermediate and near vision between EDOF IOL mixed trifocal IOL group and bilateral trifocal IOL group. However, the mixed group had better contrast sensitivity and less dysphotopsia.

In this study, we compered the full distance vision especially the near vision in micro monovision, non-micro monovision and mixed groups. Both bifocal IOL (ZMB00, + 4.00D) and EDOF IOL with some residual myopia in no-dominant eye can significantly improve the near vision. The methods of micro monovision and the mixed can both help patient obtain good distance, intermediate and near vision. However, micro monovision method has better intermediate vision and mixed method has better near vision. The non-micro monovision method obviously affects the binocular near vision. Recent studies show that the blended trifocal IOL and EDOF IOL help patients improve near visual acuity [6–7], and the blended method showed better near visual acuity than binocular Implantation of EDOF IOLs [8]. This suggest that the IOL implantation method should be considered in combination with the patient’s daily life style. For patients with high requirements for distance, intermediate and near vision, especially the near vision, the mixed method is probably advised. For patients with high requirements for distance, intermediate and near vision, especially the intermediate vision, the micro monovision method may be better. For patients with high requirements only for distance, intermediate vision, the non-micro monovision method is optional.

EDOF IOL has better contrast sensitivity and less serious dysphotopsia than other presbyopia correction IOLs, especially multifocal IOLs [17–18]. The research of B ö HM et al. [19] found that compared with other presbyopia correction IOLs, EDOF IOL has similar probability of dysphotopsia, but the severity is lighter. Our results show that the eyes implanted with Symfony IOL perform better in contrast sensitivity, MTF and PSF than those implanted with ZMB00 IOL. At the same time, the subjective dysphotopsia of the patients was also less in the micro monovision group and the non-micro monovision group than in the mixed group. This is consistent with previous research results. These findings again suggest that if patients have high requirements for postoperative contrast sensitivity and require good distance, intermediate and near vision, such as professional painters and photographers, micro monovision method may be adopted better. The study of Zhu et al. [9] showed that the blended implantation of EDOF and bifocal IOL had good near vision and slight photic disturbance and bilateral implantation of EDOF IOLs had better visual quality. This is consistent with our research results. However, their study did not compare the stereoscopic function, while our study showed that there was no significant difference in stereoscopic function among the three groups.

Presbyopia correction IOL can help patients achieve spectacle independence, but it cannot achieve complete spectacle independence at all distances. In the previous studies of implantation of Symfony IOL in both eyes using micro monovision method, the results showed that the glasses demand was between 10% and 16% [20–22]. Meanwhile ZMB00 IOL had a glasses demand for near distance rate of about 4-12% [23–26]. According to the results of our study, almost all patients can achieve spectacle independence in the distance, intermediate and daily near tasks. However, in completing the meticulous near tasks, such as needle threading and reading the medication instructions, the micro monovision group only has a 47.3% to achieve spectacle independence, and the non-micro monovision group has a lower rate, only 40%. Even in the mixed group with the best near vision, only 57.3%. This result reflects the limitations of presbyopia corrected IOL. Therefore, it is very important to have good communication before the operation to improve the satisfaction of patients after the operation [27].

The optimal refractive target for EDOF intraocular lenses in both dominant and non-dominant eyes to achieve better binocular vision in all ranges after cataract surgery continues to be discussed. The study of Jackson et al. [28] found that the optimal refractive target was − 0.08D in the dominant eye and − 0.63D in the nondominant eye to achieve good visual outcomes at all distances. Other studies suggested that too much residual myopia(>1.50D) may influence the stereoscopic function [29–32]. In order to understand more details on the best refractive target for full vision in our patients, we performed a sub-group analysis in micro monovision group, i.e., subgroup of -0.5—0.75D vs. that of -0.75 — -1.00D. Our findings showed that there is no statistical difference between the two sub-groups of micro monovision in pupil size, corneal astigmatism and spherical aberration. There was a tendency for the − 0.75 ~ -1.0D subgroup to have better uncorrected binocular near vision than the − 0.5 ~ -0.75D subgroup. In addition, the binocular stereopsis was not affected significantly. Although limited by the small number of subgroups, we can surmise that more residual myopia up to -1.00D may be beneficial to the improvement of near vision and will not affect the binocular distance vision and stereoscopic function. This is especially important for myopic patients who care more about near vision after cataract surgery. It should be noted that different ethnic groups, arm length and reading habits also affect the choice of residual myopia. How to set the most proper target for residual myopia according to patient requirements still needs further clinical exploration.

As also shown in Jackson’s study, 17 of their patients achieved full distance visual acuity with the refractive targets of − 0.07D for the dominant eye and − 0.21D for the nondominant eye. The authors speculated that this might be related to patients’ age, astigmatism, pupil size and the high-order aberration of the cornea. Indeed, age, corneal astigmatism, pupil diameter, corneal spherical aberration and IOL centrality will all affect the patient’s depth of field, thus may play a role in the postoperative visual acuity across all distances [33]. In the subgroup analysis of the non-micro monovision group, it can be seen that although the two subgroups have no statistical difference in corneal astigmatism, corneal spherical aberration and pupil diameter, the younger patients have better near vision. This may be related to pseudoaccommodation and stronger neural adaptation in younger patients. It is noteworthy that, in the subgroup with better near visual acuity, the non-dominant eye retained 0.2D more myopia, whether this plays a role in improving the near vision in younger patients needs more observations.

## Conclusion

Both micro monovision and mix-and-match methods can help patients to obtain better visual outcomes at different distances. Non micro monovision methods will affect patients near vision outcomes. We have realized the limitations of this study of the relatively small number of patients and short follow-up time, further studies are needed especially with the regards of questions such as exploring the optimum refractive target in non-dominant eyes and other factors affecting visual acuity across all distances. Since EDOF IOL has better contrast sensitivity and less degree of dysphotopsia but relatively poor near vision, how to make best use of the advantages and bypass the disadvantages should be a key consideration in the surgical plan., Personalized design is of particular importance.

## Data Availability

The datasets used and/or analyzed in the current study are available from the corresponding author upon reasonable request.

## List of abbreviations

IOL: Intraocular lens
EDOF: Extended depth of focus
CDVA: Corrected distance visual acuity
UDVA: Uncorrected distance visual acuity
UIVA: Uncorrected intermediate visual acuity
UNVA: Uncorrected near visual acuity
OCT: Optical coherence tomography
MTF: Modulation Transfer Function
PSF: Point Spread Function
